# The impact of chemotherapy on frontal lobe executive functioning in adolescents and young adults diagnosed and treated for blood cancers: a systematic review

**DOI:** 10.1186/s12885-026-16731-7

**Published:** 2026-08-15

**Authors:** Raina Patwardhan, Clare Jacobson, Jaishibani Mande Nagarajan, Lee D Hudson

**Affiliations:** 1 Great Ormond Street UCL Institute of Child Health, London, UK; 2 Guy’s and St Thomas’ NHS Foundation Trust, London, UK; 3 Peninsula Medical School, University of Plymouth, Plymouth, UK

**Keywords:** Chemotherapy, Executive function, Young adults, Blood cancers, Cognitive impairment, Systematic review

## Abstract

**Background:**

Cancer-related cognitive impairment (CRCI), often termed “chemobrain,” is a recognised effect of cancer therapy. However, most research has focused on older adults with solid tumours, leaving the neurocognitive consequences of chemotherapy in adolescents and young adults (AYAs) with blood cancers poorly understood. Because executive functions (EF)—including working memory, inhibition, flexibility, planning, and processing speed—continue to mature into early adulthood, this group may be particularly vulnerable to neurotoxicity.

**Methods:**

Following PRISMA 2020 guidelines, a systematic search was conducted in PubMed, PsycINFO, Embase (including Embase Classic), MEDLINE, and Scopus. Eligible studies included participants aged 16–25 years at diagnosis of a haematological malignancy who had received chemotherapy and were assessed for EF outcomes using validated measures. Methodological quality was appraised with an adapted Newcastle–Ottawa Scale (NOS).

**Results:**

Twelve studies met inclusion criteria. Evidence indicated consistent vulnerability in working memory and processing speed, with less consistent findings for inhibition, flexibility, and planning. Heterogeneous methods, small samples, and frequent reliance on self-report limited comparability. High-quality, controlled studies employing objective tests reported measurable EF deficits following chemotherapy.

**Conclusions:**

Chemotherapy may compromise EF in AYAs with blood cancers, though the evidence base remains methodologically limited. Standardised EF assessment, longitudinal research with baseline data, and integration of cognitive monitoring into survivorship care are recommended.

**Supplementary Information:**

The online version contains supplementary material available at https://doi.org/10.1186/s12885-026-16731-7.

## Introduction

Advances in cancer detection and treatment have significantly improved survival rates among adolescents and young adults (AYAs), broadly defined as individuals aged 15–39 years, diagnosed with haematological malignancies in the last two decades [1]. However, although survival has increased, concern has grown for longer-term physical, psychological, and neurocognitive consequences of cancer treatments. One of the most commonly reported sequelae is cancer-related cognitive impairment (CRCI), often referred to as “chemo brain” or “brain fog” [2].

CRCI encompasses deficits in attention, memory, processing speed, and higher-order executive functions (EF) [2, 3]. Adolescence and young adulthood constitute a key period for neurodevelopment, during which the prefrontal cortex and associated executive networks in particular undergo structural and functional maturation. Processes such as synaptic pruning, myelination, and the refinement of cortico-subcortical connectivity persist well into the late twenties [4, 5]. This stage supports the consolidation of complex executive abilities; working memory, inhibition, cognitive flexibility, planning, and processing speed, that underpin self-regulation, academic performance, and adaptive decision-making. Consequently, any insult to this developmental trajectory may have enduring implications for cognitive efficiency and psychosocial adjustment in AYAs.

Chemotherapeutic agents used in blood cancers, including methotrexate, cytarabine, and doxorubicin, readily cross the blood–brain barrier and are associated with neuroinflammation, oxidative stress, and altered white-matter integrity [6, 7]. Such changes may compromise prefrontal efficiency and connectivity at a time when these neural systems are still undergoing refinement. Because adolescence and young adulthood involve ongoing myelination, synaptic reorganisation, and consolidation of executive networks, chemotherapy-related neurotoxicity has the potential to alter typical developmental trajectories, potentially leading to inefficiencies or delays in the maturation of prefrontal circuits [8, 9].

To our knowledge, a systematic synthesis of the literature on the impact of chemotherapy on executive function in AYAs diagnosed and treated with blood cancers has never been conducted. We therefore carried out a systematic review of published data on the impact of blood cancer and associated treatment on executive function in AYAs, using PRISMA guidelines. For the purpose of this review, we utilized an age range of 16–25 years to define the AYA population, in keeping with one commonly established framework [1, 3]. This specific age bracket is highly distinct as it reflects the predominant prevalence of blood cancers within this demographic [1–4], while simultaneously representing a critical, vulnerable window for neuropsychological development [2, 3]. Synthesizing the data within this precise framework is urgently needed to inform clinical practice, facilitate the identification of vulnerable cognitive domains, and support the development of targeted assessment and neurocognitive intervention strategies. Ultimately, this synthesis aims to identify current methodological limitations in the literature and highlight essential avenues for future research.

## Methods

This review followed PRISMA 2020 reporting standards and was preregistered with PROSPERO (CRD420251011702).

### Eligibility criteria

Eligibility criteria were defined using a modified PICOS framework:

- Population: AYAs aged 16–25 years at diagnosis with a haematological malignancy (leukaemia, lymphoma, or myeloma).
- Intervention: Chemotherapy as a primary treatment component; studies including radiotherapy were eligible unless whole-brain radiotherapy was administered.
- Comparator: Pre/post-treatment or non-chemotherapy control groups.
- Outcomes: Executive-function performance measured by validated objective tests (e.g., Trail Making Test, Stroop, Digit Span) or psychometrically sound self-report tools (e.g., BRIEF-A, FACT-Cog).
- Design: Quantitative empirical studies in English, published in peer-reviewed journals.

### Search strategy

We searched across PubMed, PsycINFO, Embase (including Embase Classic), MEDLINE, and Scopus, from January 2025 back to database inception, combining controlled vocabulary and free-text terms related to “young adults,” “blood cancer,” “chemotherapy,” and “executive function.” Reference lists of included papers were hand-searched to identify additional studies.

**Fig. 1:**
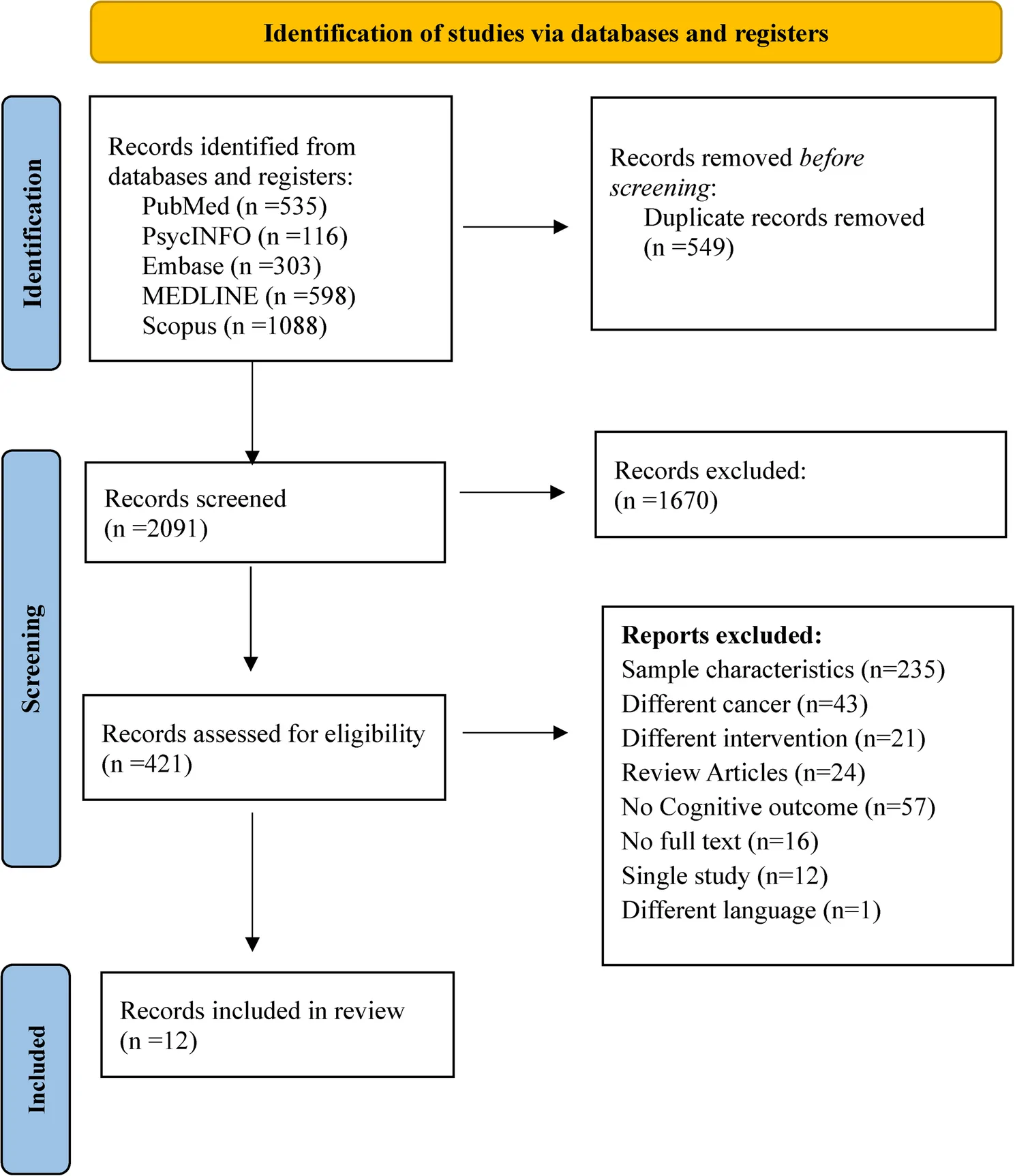
PRISMA flow diagram

A breakdown of the number of records retrieved from each individual database is provided in the PRISMA chart (Fig. 1). The final list of search terms structured by population, intervention, and outcome will be provided in Appendices I, II, and III. All stages of the search and screening process were independently verified by two researchers (RP and JMN). No disagreements for inclusion occurred.

### Study selection

Titles, abstracts, and full texts were screened against the eligibility criteria. Screening decisions were independently confirmed by two researchers (RP and JMN), with complete agreement at all stages.

### Data extraction

Data extraction was conducted by one researcher (RP), with full, independent cross-verification by a secondary researcher (JMN) to minimise selection and extraction bias. Extracted variables included study characteristics (sample, design, cancer type, treatment details), executive-function measures, and reported outcomes, which were compiled and tabulated (see Appendix IV).

### Quality appraisal

Methodological quality was assessed using the Newcastle–Ottawa Scale (NOS), adapted for observational designs [10]. Quality ratings were independently reviewed and validated by a second researcher (JMN). Studies scoring ≥ 7 were classified as high quality, 5–6 as moderate, and < 5 as low quality.

Due to insufficient data to justify a meta-analysis, findings are reported as a narrative synthesis, organised by executive-function domain (working memory, inhibition, cognitive flexibility, planning, and processing speed).

## Results

Twelve studies [11–23] met inclusion criteria, representing ~ 7,000 AYAs with leukaemia, lymphoma, or mixed haematological malignancies. Most employed cross-sectional designs; one study was longitudinal [16]. 6 of the included studies compared cases with controls [11–13, 15, 18, 23] and chemotherapy regimens varied (e.g., ABVD, BEACOPP, methotrexate-based). Cognitive assessments ranged from neuropsychological batteries to validated self-report questionnaires. Included studies are summarised in Table 1.

**Table 1:** Summary characteristics of included studies

Study	Control(*n*)	Sample	Gender	Cognitive Tests	Cognitive Findings
Krull et al. 2012 [12]	Population Norms (Age adjusted)	62	Male = 29 (46.8%), Female = 33 (53.2%)	WASI, Trail Making Test, Conners’ Continuous Performance Test-II, Digit Span Forward of WAIS-III, California Verbal Learning Test-II, Groove Pegboard, Stroop Colour Word Test	Survivors demonstrated poor sustained attention (*P*=.004), variability of attention (*P*=.01), and attention span (*P*=.01). Short-term memory recall was below expectations (*P*=.001), as was long-term recall (*P*=.006).
Doolittle et al. 2013 [17]	NR	80	Male = 43 (54%), Female = 37 (46%)	WASI, Digit Span, Trail Making Test, Hopkins Verbal Learning Test	47% in WBRT group impaired in executive function, attention, and memory domains. Women had a mean score 0.31 points (95% CI, 0.01–0.62) lower than men in the attention and executive function domain.
Prasad et al. 2015 [13]	390 siblings (adjusted for age and sex)	2,589 AYA survivors	Male = 1284 (49.6%), Female = 1305 (50.4%)	CCSS Neurocognitive Questionnaire (CCSS-NCQ)	Reported more cognitive symptoms than siblings; high executive function complaints. Survivors reported significantly greater emotional distress—anxiety (OR = 2.00), somatization (OR = 2.36), and depression (OR = 1.55).
Nugent et al. 2018 [15]	*n* = 14 (age and gender-matched healthy controls)	23	Male = 16 (69.6%), Female = 7 (30.4%)	Digit Vigilance Test, Digit Symbol Substitution Test, Grooved Peg Board Test, Stroop Color and Word Test, Verbal Fluency Test, Trail Making Test, Wechsler Memory Scale, Letter Number Sequencing Test	No significant objective cognitive deficits; subjective cognitive complaints reported. There were no significant differences in cognitive function scores between cancer survivors and controls across most tests.
Trachtenberg et al. 2018 [11]	*n* = 14 (age and education matched healthy controls)	51	Male = 24 (47%), Female = 27 (53%)	California Verbal Learning Test (CVLT), Digit Span subtest (WAIS-III-R), Trail Making Test, Stroop Test, Raven Progressive Matrices, CANTAB Paired Associates Learning (PAL), CANTAB	Executive function most affected (41% impairment); fatigue and anxiety were contributing factors. Survivors reported significantly more frequent and severe subjective experiences of cognitive impairment and fatigue compared to controls, as measured by the FACT-Cog and MFI-20 respectively.
Tan et al. 2020 [16]	NR	91	Male = 49 (53.8%), Female = 42 (46.2%)	Functional Assessment of Cancer Therapy-Cognitive Function (FACT-Cog)	34.1% reported CRCI, notably executive deficits.
Dewar et al. 2021 [18]	228,029 (no history of cancer control group)	2,646 AYA survivors	Male = 486 (18.4%), Female = 2160 (81.6%)	NHIS Functioning and Disability Questionnaire	Higher cognitive dysfunction prevalence in cancer survivors (24.8%) vs. controls (15.3%).
Mariegaard et al. 2021 [14]	NR	116	Male = 58 (50%), Female = 58 (50%)	Behavior Rating Inventory of Executive Function-Adult (BRIEF-A)	39% showed executive impairments; self-reported difficulties with organization. Survivors reporting impaired executive functioning reported worse mental health (ps < 0.001) than survivors reporting non-impaired executive functioning
Mellblom et al. 2021 [22]	NR	359	Female = 165 (45.9%), Male = 194 (54%)	NORCAYACS questionnaire	Non-Hodgkin survivors reported highest cognitive symptom burden (33% reported 5 + late effects). 33.3% of NHL and 35.1% Leuk survivors reported concentration and memory problems
Brock et al. 2022 [20]	NR	502	Male = 127 (25.3%), Female = 375 (74.7%)	Copenhagen Psychosocial Questionnaire, COPSOQ	Approximately 20% of AYA cancer survivors reported cognitive impairments at both timepoints (t1 and t2), indicating that these difficulties are persistent over time [*n* = 88 (18%) reported CRCI at t1 and *n* = 82 (16%) at t2].
Baratto et al. 2024 [21]	NR	10	NR	WASI-II, Wide Range Assessment of Memory and Learning, Second Edition (WRAML-2), Delis–Kaplan Executive Function System (D-KEFS)	9 of 10 had at least one negative neurocognitive outcome (mean no. of impaired tests = 3; range = 1–7); frontal executive issues.
Williams et al. 2024 [23]	199 (age, sex and race/ethnicity frequency-matched community controls)	197	Male = 95 (48.2%), Female-102 (51.8%)	Woodcock-Johnson III Tests of Achievement, Trail Making Test, Conners’ Continuous Performance Test, WASI, Wechsler Adult Intelligence Scale, CVLT	HL survivors had significantly poorer executive function than controls across multiple domains.

### Categorization of cognitive domains

To provide a clear and structured synthesis of the evidence base, neurocognitive outcomes are categorized below by specific functional domains. First, foundational cognitive resources, specifically information processing speed and general attention, are evaluated, as these baseline skills underpin higher-order cognition. Following this, findings relating to discrete core executive function domains are examined individually, including working memory, inhibitory control, cognitive flexibility, and planning. Finally, subjective patient-reported outcomes (self-report data) are synthesized separately to distinguish perceived everyday functional difficulties from performance-based objective neuropsychological testing.

### Working memory

Working memory was the most frequently assessed domain. Objective data from studies showed significantly poorer performance on Digit Span and Letter Number Sequencing relative to controls, indicating reduced short-term storage and manipulation capacity [11, 12].

In contrast, Nugent et al. [15] which included a smaller AYA sample and used a broader cognitive battery, did not find significant differences in working memory or related subtests between cancer survivors and healthy controls. Overall, converging evidence points to working-memory vulnerability post-chemotherapy. The authors, however, did acknowledge that this null result might reflect sample size limitations, missing pre-treatment baselines or the relatively short time since treatment (less than 3 years for most participants).

### Attention and vigilance

Regarding basic, non-executive attentional capacity, survivors demonstrated significant vulnerabilities on timed vigilance tasks. In the study by Krull et al. [12], survivors, in comparison to age-adjusted population norms, demonstrated poor sustained attention (*P*= .004), heightened semantic variability of attention (*P*= .01), and a significantly reduced baseline attention span (*P*= .01).

### General and long-term memory

Generic non-executive memory storage and retrieval systems showed notable vulnerabilities following treatment. In the objective cohort evaluated by Krull et al. [12], basic short-term memory recall was found to be significantly below standardized expectations (*P*= .001). Furthermore, long-term episodic and delayed recall metrics were similarly impaired (*P*= .006) relative to normative expectations.

### Inhibitory control

Findings for inhibitory control were inconsistent. Krull et al. [12] identified deficits on specialized objective inhibition tasks, whereas Nugent et al. [15] reported intact performance. Within the Nugent et al. [15] cohort, a small effect size was observed specifically on the Stroop Test (d= -0.226), which serves as a standard neuropsychological metric of response inhibition.

Doolittle et al. [17] assessed cognitive outcomes using subtests from the Wechsler Adult Intelligence Scale and related tools, and reported selective impairments in inhibition among participants who had received whole-brain radiation therapy (WBRT) in addition to chemotherapy i.e., in the WBRT group, 47% were impaired in multiple domains, compared with 9% in the high-dose methotrexate–based group, 16% in the BBBD group, and 13% in the high-dose chemotherapy and ASCT group. However, the findings were not stratified by treatment type, making it difficult to isolate the effect of chemotherapy specifically. Moreover, the study’s focus on older adults and its wide age range (10 to 78 years) limits generalisability to the AYA subgroup.

### Cognitive flexibility

Objective cognitive flexibility, assessed via the Wisconsin Card Sorting Test (WCST) and Trail Making Test Part B (TMT-B), was modestly impaired in several studies [12, 15, 17]. Survivors exhibited longer completion times and reduced accuracy, reflecting distinct set-shifting difficulties.

Of the included studies, Nugent et al. [15] explicitly utilised the WCST, a widely accepted gold-standard measure of set-shifting. Their results showed no statistically significant impairment in cognitive flexibility among chemotherapy-treated young adult survivors when compared directly to healthy controls (*p* = .715). However, the study authors noted that the small sample size and relatively short time since treatment (2 or more years) may have limited the empirical detection of more subtle deficits.

Krull et al. [12] and Doolittle et al. [17] both reported difficulties in tasks requiring active shifting and divided attention using the TMT-B. In these studies, survivors exhibited significantly longer completion times and higher error rates on the TMT-B compared to normative data or internal benchmarks. However, neither study used a cancer-free control group, limiting the ability to isolate chemotherapy-specific effects.

### Planning and organisation

Planning was rarely measured directly in the current literature base. Trachtenberg et al. [11] included subtests from the automated CANTAB battery, such as the Stockings of Cambridge (SOC) task, which evaluates objective planning and problem-solving. Their results indicated clear impairments in planning ability, with highly statistically significant findings (*P* < .0001). The study also noted that longer time since treatment was associated with more pronounced planning difficulties. Overall, while objective difficulties are explicitly documented, the lack of widespread testing with tools like the SOC means the performance evidence base remains tentative and underdeveloped.

### Subjective patient-reported outcomes (Self-Report Questionnaire Data)

Across the literature, large-scale self-report studies [13, 14, 16, 18] found heightened daily complaints regarding perceived executive difficulties, which frequently diverged from objective performance testing.

Regarding working memory and multitasking, Prasad et al. [13] demonstrated that AYA survivors reported significantly more daily neurocognitive problems compared to siblings, particularly in task efficiency (OR = 1.72), emotional regulation (OR = 1.74), and general memory (OR = 1.44), after adjusting for age and sex. Similarly, Mariegaard et al. [14] relied on the Behavior Rating Inventory of Executive Function (BRIEF-A) and found that 39% of survivors reported clinically significant executive impairment, with subjective working memory identified as the single most frequently reported deficit (24%).

Self-report instruments evaluated by negative symptom checklists [16] revealed high levels of perceived impulsivity and poor concentration. Overall, 34.1% of participants reported chemotherapy-related cognitive impairment at least once during the study period, endorsing persistent problems with daily concentration on the Distress Thermometer (DT) checklist.

Furthermore, questionnaires like the CCSS-NCQ and BRIEF-A identified cognitive flexibility as a prominent area of subjective difficulty, particularly regarding multitasking and adapting to changes in daily life [14, 16, 18]. Nugent et al. [15] noted that differences in total perceived cognitive function scores exhibited a small to medium effect size (d = 0.441), indicating that survivors (M = 32.78, SD = 23.02) reported greater daily difficulty with overall functional flexibility compared to healthy controls (M = 23.71, SD = 15.54).

### Processing speed (Foundational Skill)

In addition to core executive functioning domains, data on processing speed was synthesized. While recognized theoretically as a foundational cognitive skill rather than a core executive function, it was analyzed alongside executive outcomes due to its critical role as an underlying mechanism supporting higher-order executive performance.

Processing speed emerged as the most consistently affected domain across the review. Trachtenberg et al. [11], one of the few high-quality studies utilizing a matched control group, reported statistically significant reductions (*P* < .001) in processing speed using timed tasks such as the Trail Making Test Part A (TMT-A). Additionally, Nugent et al. [15] observed small to moderate negative effect sizes on the Digit Vigilance Test (d = 0.396), indicating subtle reductions in baseline tracking speed. These processing impairments were particularly notable in survivors treated with high-intensity chemotherapy regimens.

Krull et al. [12] also found significantly slowed processing speed (*P* = .03) in their Hodgkin lymphoma survivor sample, assessed via timed attention and vigilance tasks. Self-reported findings strongly supported these results, with AYAs frequently describing profound mental slowness and difficulty keeping pace with daily cognitive demands [13, 14, 18].

### Quality appraisal findings

Overall methodological quality ranged from low to high, with most studies rated as moderate on the adapted Newcastle–Ottawa Scale. A minority achieved high-quality ratings, typically those employing clearer comparator groups and standardised neuropsychological assessments. Across included studies, common methodological limitations included small sample sizes, reliance on self-report measures, and heterogeneity in executive-function assessments. The absence of pre-treatment baselines in most studies further restricted the ability to infer treatment-related cognitive change.

## Discussion

Overall, approximately half of the included studies documented objective neuropsychological performance deficits, which were predominantly concentrated within the domain of working memory. Similarly, widespread vulnerabilities were identified in processing speed, highlighting the prominent impact of chemotherapy on this foundational cognitive skill. In contrast, performance metrics for higher-order executive functions- such as inhibitory control, cognitive flexibility, and planning- yielded highly inconsistent or underdeveloped objective data. Across the literature base, subjective, patient-reported impairments were significantly more prevalent than objective performance deficits. Ultimately, while the widespread heterogeneity of methodologies and the systemic absence of pre-treatment baselines hindered a formal quantitative synthesis, the evidence collectively supports a distinct chemotherapy-associated executive and foundational cognitive vulnerability in AYA blood cancer survivors.

To our knowledge, our review provides the first focused synthesis of chemotherapy-related executive-function (EF) outcomes in adolescents and young adults (AYAs) with blood cancers. Together, these findings raise the possibility that disruption of frontal-subcortical circuits and white-matter integrity may contribute to observed EF changes. Working memory and processing speed are heavily dependent on prefrontal networks and their connectivity with subcortical regions, functions that are particularly sensitive to diffuse white-matter disruption [6–9].

Neuroimaging studies in cancer populations have reported chemotherapy-associated alterations in prefrontal activation and white-matter microstructure, including reduced integrity within frontal tracts linked to executive performance [6, 19]. These established neural alterations are highly consistent with the prominent, pervasive deficits in processing speed observed across our included cohorts, which align with neuroimaging evidence of chemotherapy-related white-matter disruption and diffuse leukoencephalopathy. Importantly, from a theoretical standpoint, cognitive models generally conceptualize processing speed as a foundational, lower-level cognitive skill that supports and mediates higher-order executive control, rather than being synonymous with an executive function itself. The prominent processing speed deficits observed across these studies suggest that apparent executive dysfunctions in AYA blood cancer survivors may be partially driven or exacerbated by a slowed rate of information processing, rather than representing an isolated breakdown in executive control mechanisms. However, no neuroimaging studies to date have examined these mechanisms specifically in AYAs with blood cancers, and direct investigation is therefore required to determine whether chemotherapy-related EF impairment in this population reflects disruption of these developing neural systems.

The magnitude, onset, and persistence of EF dysfunction varied across studies, suggesting that observed impairments are shaped by multiple interacting influences, including individual vulnerability, treatment characteristics, and methodological factors, rather than reflecting a uniform cognitive profile. Working-memory and processing-speed deficits were the most consistently reported, whereas findings for inhibitory control, cognitive flexibility, and planning were mixed. This inconsistency may reflect several sources of bias: (1) heterogeneity in measurement, as few studies employed gold-standard, domain-specific tasks; (2) time-dependent effects, whereby certain EF components may recover more rapidly than others; and (3) variability in samples and treatments, including differences in chemotherapeutic agents, cumulative dose, central nervous system prophylaxis, and adjunctive radiotherapy.

The patterns we found mirror those in older adult CRCI research, which frequently implicates working memory and processing speed as the most affected domains [2, 3], yet the different developmental context distinguishes AYAs from adults and necessitates a separate consideration. Ongoing prefrontal maturation, synaptic pruning, and myelination render the brain simultaneously adaptable and susceptible to injury compared to older adults [4]. Neurotoxic disruption during this stage may impede maturation trajectories, leading to enduring inefficiencies even after treatment cessation.

While neurocognitive outcomes in childhood and pediatric cancer survivors have been extensively documented over recent decades (e.g., Cheung & Krull, 2015), the distinct lack of focused research within isolated AYA cohorts represents a critical gap that this review explicitly addresses. It is clinically problematic to extrapolate pediatric findings to the AYA population, as AYAs are situated at a fundamentally unique point of neuropsychological, biological, and psychosocial development. Neurologically, the AYA years are characterized by rapid, late-stage maturation of frontal executive circuits, intensive synaptic pruning, and post-pubertal hormonal shifts that alter neurobiological vulnerability and plasticity compared to younger children. Psychosocially, the cognitive demands placed on an AYA are vastly different from those of a pediatric patient; AYAs are navigating complex, high-stakes life transitions- such as tertiary education, independent living, financial management, and early career entry- which place immense strain on emerging executive control networks. Consequently, studying AYAs independently as a distinct clinical entity is vital, as the interaction between neurotoxic treatments and this specific developmental window yields a unique cognitive survivorship profile that pediatric models cannot accurately predict.

Available evidence suggests several plausible moderators that can explain heterogeneity: age at treatment within the AYA range; treatment intensity and specific agents—which likely drive the dose-related neurotoxic effects observed in foundational processing speed profiles; time since treatment—which may reflect delayed or cumulative neurotoxic effects on planning and structural executive performance over time; cognitive reserve (education, premorbid IQ); and psychosocial factors (fatigue, depression, stress) [2, 3, 6, 10, 19].

Our review had several strengths and limitations. We conducted a systematic and comprehensive search of the literature, with screening and verification completed by two independent researchers, and we appraised study quality using an established, validated tool. However, the studies identified were constrained by several recurring methodological weaknesses. Most were based on small, heterogeneous samples and frequently employed cross-sectional designs, which impede the ability to examine the onset, progression, or recovery of executive-function difficulties over time. A particular limitation was the scarcity of pre-treatment cognitive baselines, which hampers causal inference and prevents clear disentanglement of disease- versus treatment-related effects. Case-control methodologies were also absent in the majority of studies. Executive-function measures varied considerably across the evidence base, ranging from brief screening tools to full neuropsychological batteries, limiting comparability. Many studies relied heavily on subjective questionnaires without corroborating objective cognitive data. Crucially, empirical evidence demonstrates that self-reported measures and performance-based objective neuropsychological tests often capture entirely distinct constructs; objective tasks evaluate isolated, optimal cognitive capacity under structured testing conditions, whereas self-reports are heavily influenced by an individual’s meta-cognition, mood, fatigue, and real-world functional demands. Furthermore, our review is limited by the exclusion of informant or “other-reports” (such as parent, partner, or clinician ratings). This represents a distinct gap, as comparing self-reports with other-reports can provide valuable insight into a patient’s insight and everyday executive dysfunction. Furthermore, key confounding variables, such as education, mood, sleep, pain, and corticosteroid exposure, were inconsistently measured or controlled for, reducing the precision of reported associations.

Additionally, we acknowledge that a prominent limitation of the current evidence base is diagnostic heterogeneity, as several large-scale studies reported findings from mixed cancer cohorts where blood cancer outcomes could not be statistically isolated. While completely excluding these studies would have severely depleted an already scarce AYA dataset and omitted substantial numbers of blood cancer survivors embedded within them, their inclusion necessitates caution when generalizing findings. Mechanistically, the validity of our broader conclusions is supported by shared treatment pathways, as non-blood-cancer patients within these cohorts underwent highly similar neurotoxic chemotherapeutic regimens known to affect central nervous system white matter. Nonetheless, to prevent over-generalization, we have explicitly noted throughout our Results section where findings rely on these mixed diagnostic samples, and we issue a formal call for future AYA neurocognitive research to utilize stratified, disease-specific diagnostic arms. At the review level, we also acknowledge that the definition of the AYA population varies internationally. Whilst we utilized a strict inclusion cutoff of 16–25 years (as outlined in our introduction and PROSPERO protocol) to capture a specific developmental window, the findings in our study may not fully relate or generalize to clinical groups where a broader categorization (e.g., 16–40 years) is used.

### Clinical and research implications

Our findings highlight a substantial gap in the robust, methodologically sound investigation of chemotherapy-related cognitive outcomes in AYAs with blood cancers. Critically, no studies to date have conducted pre- and post-treatment cognitive assessments in this population, representing a major limitation in the field. Future research should prioritise baseline (pre-treatment) testing, immediate post-treatment assessment, and long-term follow-up, in order to determine whether cognitive functioning recovers over time, whether development that was disrupted during treatment later “catches up” or whether executive difficulties persist as enduring deficits. Such data is essential not only for characterising cognitive trajectories, but also for informing the timing, nature, and intensity of cognitive or psychosocial interventions required to support rehabilitation and functional reintegration during survivorship.

The apparent differences between objective and self-reported impairment underscores the importance of multimodal assessment (objective tests, validated patient-reported outcomes and ecological measures) incorporating both perspectives to capture a fuller clinical picture. In clinical settings, routine neurocognitive monitoring should be integrated into AYA oncology care pathways, ideally anchored to pre-treatment baselines. The use of standardised, developmentally appropriate executive-function batteries, administered longitudinally, would enable clearer identification of emerging difficulties and more accurate tracking of developmental trajectories. Crucially, knowledge of common EF impacts and their trajectories must be translated into proactive survivorship care and comprehensive cognitive rehabilitation strategies. Rather than relying solely on individual remediation, interventions should ideally be delivered within a systemic family therapy approach to support both patients and their support systems. For example, most UK AYA cancer services provide targeted, multidisciplinary ‘end of treatment’ education days for patients and families. Including specialist neuropsychological and family therapy advice as a standard component of these sessions would provide practical techniques for how to proactively adjust, implement educational/vocational scaffolding, and adapt daily environments to account for the cognitive impacts of treatment. While current evidence for targeted pharmacological management (such as psychostimulants) in this specific age cohort remains scarce, validating the real-world effectiveness of these holistic cognitive and psychosocial interventions could establish them as a minimum standard in clinical guidelines for AYA cancer care.

Future research should also integrate cognitive assessment with neuroimaging and biomarker data to clarify underlying neurobiological mechanisms, while examining potential moderators, including treatment intensity, corticosteroid exposure, emotional symptoms, sleep, and psychosocial stress, that may shape vulnerability or resilience. Together, these steps would help align clinical practice with the developmental and neurobiological needs of AYAs and significantly strengthen the evidence base for intervention.

## Conclusion

Chemotherapy appears to adversely affect neurocognitive functioning in adolescents and young adults (AYAs) with blood cancers, most notably impacting objective working memory performance alongside processing speed, which serves as a foundational cognitive skill. While widespread subjective self-reports underscore significant real-world challenges with daily multitasking and adaptability, substantial methodological constraints across the current literature base- including diagnostic mixing and a lack of pre-treatment baselines- preclude firm causal conclusions. Nevertheless, converging evidence indicates a clinically meaningful cognitive vulnerability during this critical developmental window.

Addressing these structural knowledge gaps urgently requires harmonised, prospective, and multimodal research that integrates standardized cognitive batteries with neuroimaging data. Clinically, incorporating routine neurocognitive tracking anchored to pre-treatment baselines and translating these findings into structured survivorship care, such as systemic family therapy approaches and proactive educational scaffolding, is vital. Implementing these integrated research and clinical steps will better safeguard AYAs’ long-term educational, occupational, and psychosocial outcomes, ensuring that cognitive rehabilitation becomes a minimum standard in survivorship care.

## Supplementary Information

Supplementary Material 1.

Supplementary Material 2.

## Data Availability

All data generated or analysed during this study are included in this published article [and its supplementary information files].
