# Supplementary material for: The impact of chemotherapy on frontal lobe executive functioning in adolescents and young adults diagnosed and treated for blood cancers: a systematic review
Source: BMC Cancer. 2026 Aug 15;26:1401. doi: 10.1186/s12885-026-16731-7 (PMC13753297; doi:10.1186/s12885-026-16731-7)
Supplement: Supplementary file 1 [file 12885_2026_16731_MOESM1_ESM.docx]

**Appendix**

**Appendix I – Blood Cancer Diagnoses and Search Terms**

**Blood Cancer Diagnoses:**

- Lymphoma: Hodgkin’s lymphoma, Non-Hodgkin’s lymphoma, Burkitt’s lymphoma
- Leukaemia:
  - Acute Myeloid Leukaemia (AML)
  - Acute Lymphoblastic Leukaemia (ALL)
  - Chronic Myeloid Leukaemia (CML)
  - Chronic Lymphocytic Leukaemia (CLL)
  - Acute Promyelocytic Leukaemia (APL)
  - Hairy Cell Leukaemia (HCL)
  - Large Granular Lymphocytic Leukaemia (LGL)
  - T-cell Acute Lymphoblastic Leukaemia (T-ALL)
  - Chronic Myelomonocytic Leukaemia (CMML)

**Search Terms Used:**

- Hematologic Neoplasm
- Neoplasm, Hematologic
- Hematologic Malignancies
- Hematologic Malignancy
- Hematological Malignancies
- Hematological Malignancy
- Malignancy, Hematological
- Hematological Neoplasms
- Hematological Neoplasm
- Neoplasm, Hematological
- Malignancies, Hematologic
- Malignancy, Hematologic
- Blood Cancer
- Blood Cancers
- Cancer, Blood
- Neoplasms, Hematologic
- Hematopoietic Neoplasms
- Hematopoietic Neoplasm
- Neoplasm, Hematopoietic
- Neoplasms, Hematopoietic
- Hematopoietic Malignancies
- Hematopoietic Malignancy
- Malignancy, Hematopoietic

**Appendix II – Eligible Chemotherapy Agents, Regimens, and Search Terms**

**Chemotherapy Regimens:**

- ABVD:

A: Adriamycin (also known as doxorubicin or hydroxydaunorubicin)

B: Bleomycin

V: Vinblastine

D: Dacarbazine

- eBEACOPDac / escBEACOPDac:

e/esc: Escalated dose

B: Bleomycin

E: Etoposide

A: Doxorubicin

C: Cyclophosphamide

O: Oncovin™ (Vincristine)

P: Prednisolone

Dac: Dacarbazine

**Chemotherapy Agents:**

- Mercaptopurine (6-Mercaptopurine / 6-MP)
- Cladribine (2-CdA)
- Fludarabine
- Mitoxantrone
- Etoposide (VP-16)
- Hydroxyurea
- Corticosteroids (Prednisone, Dexamethasone)
- Methotrexate (MTX / Amethopterin / Mexate)
- Azacitidine
- Decitabine
- Liposomal daunorubicin and cytarabine (Vyxeos, CPX-351)
- Cytarabine (Cytosine arabinoside / Ara-C)
- Anthracyclines: Daunorubicin (Daunomycin), Idarubicin

Search Terms Used:

- Chemotherapy
- ABVD regimen
- eBEACOPDac / escBEACOPDac
- Mercaptopurine / 6-MP
- Cladribine / 2-CdA
- Fludarabine
- Mitoxantrone
- Etoposide / VP-16
- Hydroxyurea
- Prednisone / Dexamethasone
- Methotrexate / MTX / Amethopterin / Mexate
- Azacitidine
- Decitabine
- Liposomal daunorubicin / cytarabine / Vyxeos / CPX-351
- Cytarabine / Ara-C
- Daunorubicin / Daunomycin / Idarubicin

**Appendix III – Validated Cognitive and Executive Function Measures and Search Terms**

**Cognitive Function Measures:**

- General cognitive function / IQ
- Verbal IQ
- Performance IQ
- Memory scores / indices / scaled scores
- Processing speed
- Working memory
- Visuo-spatial awareness

**Standardised Cognitive Assessment Tools:**

- Wechsler Memory Scales (WMS)
- National Adult Reading Test (NART)
- Wechsler Adult Intelligence Scale (WAIS / WAIS-R)
- Wechsler Intelligence Scale for Children (WISC / WISC-IV / WISC-V)
- Test of Premorbid Functioning (TOPF)
- Wechsler Preschool and Primary Scale of Intelligence (WPPSI)
- Raven’s Progressive Matrices / Raven Progressive Matrices / Ravens Progressive Matrices / Raven Test

**Executive Function Measures:**

- Behavioural Assessment of the Dysexecutive Syndrome (BADS)
- Hayling and Brixton Tests
- Delis-Kaplan Executive Function System (DKEFS)
- Stroop Test
- Wisconsin Card Sorting Test
- Trail Making Test
- Verbal Fluency Task
- Frontal Assessment Battery

**Executive Function Domains / Terms:**

- Planning
- Organising
- Inhibition
- Impulsivity
- Set shifting
- Multitasking
- Executive Controls
- Attention

**Search Terms Used:**

- Cognitive function
- IQ / Verbal IQ / Performance IQ
- Memory scores / indices / scaled scores
- Processing speed / Working memory / Visuo-spatial awareness
- Wechsler Memory Scales (WMS / WMS-IV-NL)
- Wechsler Adult Intelligence Scale (WAIS / WAIS-R)
- Wechsler Intelligence Scale for Children (WISC / WISC-IV / WISC-V)
- Wechsler Preschool and Primary Scale of Intelligence (WPPSI)
- Test of Premorbid Functioning (TOPF)
- National Adult Reading Test (NART)
- Raven’s Progressive Matrices / Raven Test
- Executive function / Executive Controls
- Planning / Organising / Inhibition / Impulsivity / Set shifting / Multitasking / Attention
- BADS / Hayling and Brixton / DKEFS / Stroop Test / Wisconsin Card Sorting Test / Trail Making Test / Verbal Fluency Task / Frontal Assessment Battery

**Appendix IV- Complete Data Extraction Table**

| **Study** | **Design** | **Subject (n)** | **Gender (%)** | **Age at Diagnosis** | **Time since diagnosis** | **Time since treatment** | **Age at evaluation** | **Control (n)** | **Type of Cancer** | **Treatment** | **Cognitive Measure used** | **Cognitive Domains Measured** | **Other measures used** | **Cognitive Outcomes** | **Other outcomes** | **Assessment Schedule** | **Limitation** |
| --- | --- | --- | --- | --- | --- | --- | --- | --- | --- | --- | --- | --- | --- | --- | --- | --- | --- |
| Krull et al. 2012 | Prospective, Cross Sectional | 62 | Male=29 (46.8%), Female=33 (53.2%) | 5.9-19 years, mean 15.1 years [SD=3.30] | 18.3-39.6 years, mean 27.1 years [SD=5.65] | NR | 34.4-55.4 years, mean 42.2 years | Population Norms (Age adjusted) | Hodkin Lymphoma | Radiation and Chemotherapy [High-dose ( >30 Gy) thoracic radiation (n=38) or lower-dose (<30 Gy) thoracic radiation combined with anthracycline (n=24)] | Wechsler Abbreviated Scale of Intelligence, Trail Making Test Part A, Conners’ Continuous Performance Test-II [CPT-II], Digit Span Forward of WAIS-III, California Verbal Learning Test-II, Groove Pegboard, Stroop Colour Word Test, Trail Making Test Part B, | **Intelligence**, **attention,** **memory**, **processing speed** and **executive function** | Behavior Rating Inventory of Executive Function-Adult version (self-report behavior ratings to assess symptoms of executive dysfunction), Self-report of fatigue symptoms (Functional Assessment of Chronic Illness Therapy [FACIT] Fatigue Scale, Brain Magnet | Survivors demonstrated poor sustained attention (P=.004), variability of attention (P=.01), and attention span (P=.01). Short-term memory recall was below expectations (P=.001), as was long-term recall (P=.006). Survivors demonstrated low performance on f |  | Neurocognitive testing was conducted during a single 2-hour visit within 3 days of the medical examinations, Brain MRI examinations were obtained within 3 days of the neurocognitive testing and cardiac and pulmonary evaluations. | Selection bias, recall biasand survivor bias, Time since treatment isn’t mentioned, no follow up |
| Doolittle et al. 2013 | Prospective, Cross sectional | 80 | Male=43 (54%), Female=37 (46%) | 10 to 78 years, median 59 years | 2 to 26 years, median interval=5.5 years | Not explicitly mentioned (minimum of 2 years after histologic diagnosis and in complete remission) | calculated estimation= 64.5 years |  | Primary CNS Lymphoma | High-dose methotrexate-based chemotherapy followed by whole-brain radiotherapy (WBRT) (n = 15).  High-dose methotrexate-based chemotherapy alone (n = 32).  High-dose methotrexate (intra-arterial)-based chemotherapy with blood-brain barrier disruption (BBBD) n=25. High-dose methotrexate followed by high-dose chemotherapy and ASCT (n=8) | Attention and executive function (Wechsler Adult Intelligence Scale—III Digit Span subtest [Digits Forward, Digits Backward], Trail Making Test Parts A and B, and Brief Test of Attention), verbal memory (Hopkins Verbal Learning Test Revised), and motor skills | Attention and executive function, Verbal Memory and Motor Skills | 1. EORTC QOL-C30: measures 5 functional scales and global QOL. 2. The EORTC QOL Questionnaire Brain Cancer Module–20: 0 assesses items such as visual disorders, communication deficit, and future uncertainty. 3. MRI/CT scans | In the WBRT group, 47% were impaired in multiple domains, compared with 9% in the high-dose methotrexate–based group, 16% in the BBBD group, and 13% in the high-dose chemotherapy and ASCT group. (Excluded data of WBRT group from the following:) **Attention/** | Women had a mean score 0.31 points (95% CI, 0.01–0.62) lower than men in the attention and executive function domain. QOL (EORTC QLQ-C30, n = 71): Better neuropsychological scores were linked to higher global QOL and improved functioning across all doma | Neuropsychological evaluation performed in 60–90 minutes in the ambulatory clinic or in the survivor’s residence or workplace. Neuroimaging was requested within 90 days of neuropsychological evaluation | No consistent pre/post-treatment cognitive or MRI baseline data across groups, follow-up timing varied. Primary CNS Lymphoma may have organic causes of cognitive impairment have not been accounted for. |
| Prasad et al. 2015 | Retrospective, Cross sectional | 2589 AeYA Survivors (excluded Pediatric survivors) | Male=1284 (49.6%), Female=1305 (50.4%) | 11–14 years (48.5%), 15–21 years (51.5%) | at least 5 years |  | 25–29 years (2.4%), 30–34 years (21.9%), >35 years (75.5%) | 390 siblings(adjusted for age and sex) | Mixed [Leukemia=461 (17.8%), HL=798 (30.8%), NHL=278 (10.7%)] | Chemotherapy=540 (20.9%), Radiotherapy=494 (19.1%), Chemo+Radiotherapy=540 (20.9%) | CCSS Neurocognitive Questionnaire (CCSS-NCQ) | self-reported neurocognitive symptoms- task efficiency, emotional regulation, plan/organization, and working memory | The Brief Symptom Inventory-18 (BSI-18) is an 18-item checklist that measures symptoms of emotional distress | AeYA survivors, in comparison to siblings reported more neurocognitive problems: task efficiency (OR = 1.72), emotional regulation (OR = 1.74), and memory (OR = 1.44), after adjusting for age and sex. Lymphoma/sarcoma survivors (diagnosed at age 15–21) re | AeYA survivors vs. siblings: AeYA survivors reported significantly greater emotional distress—anxiety (OR = 2.00), somatization (OR = 2.36), and depression (OR = 1.55). |  | results were stratified by grouping CNS tumors with leukemias and grouping lymphomas with sarcomas/bone tumors |
| Nugent et al. 2018 | Prospective, Cross sectional | 23 | Male=16 (69.6%), Female=7 (30.4%) | 15-21 years, mean 17.4 years (SD=1.9) |  | 2 or more years | 18-39 years, mean 23.8 years (median 22.6 years) | n=14 (age and gender-matched healthy controls) | Mixed [ALL=4 (17.4%), AML=1 (4.3%), HL=10 (43.4%), NHL=1 (4.3%) | NR [The Intensity of Treatment Rating (ITR-3.0) Scale was used to classify the intensity of pediatric cancer therapy according to treatment modality and stage/risk level for the patient. Based on these characteristics, the ITR assigns an intensity level f | Digit Vigilance (DV) Test, Digit Symbol Substitution Test, Grooved Peg Board Test, Stroop Color and Word Test, Verbal Fluency Test, Trail Making Test A and B, Wechsler Memory Scale, Letter Number Sequencing Test, Rey-Osterrieth Complex Figure Test, Wiscon | attention, memory, executive function, psychomotor functioning, and visual perceptual skills | **Patients Assessment of Own Functioning Inventory** (PAOFI): self-report measure assessing cognitive difficulties across five domains: memory, executive function, language, orientation, and sensorimotor ability. **Center for Epidemiological Studies-Depression** | There were no significant differences in cognitive function scores between cancer survivors and controls across most tests. Small effect sizes were observed in the Digit Vigilance (DV) Test (d = 0.396) and the Stroop Test (d = -0.226), suggesting potentia | Significant correlation between perceived cognitive function and symptoms of depression, anxiety, and fatigue. |  | no pretreatment baseline, small and unequal sample (missing control), no follow up |
| Trachtenberg et al. 2018 | Prospective, Cross sectional | 51 | Male=24 (47%), Female=27 (53%) | 18 to 50 years |  | completed therapy at least 6 months and up to 5 years prior to their evaluation | 19 to 48 years, median=28 years [<25 years (31%), 25-34 years (41%), 35+ years (28%)] | n=14 (age and education matched healthy controls) | Hodkin Lymphoma | Chemotherapy=39 (76%), Chemo+Radiation=12 (24%) | California Verbal Learning Test (CVLT), Digit Span subtest (WAIS-III-R), Trail Making Test A & B (TMT), Stroop Test, Raven Progressive Matrices, CANTAB Paired Associates Learning (PAL), CANTAB Stockings of Cambridge (SOC), CANTAB Intra-Extra Dimensional S | General Intelligence Attention Processing Speed Memory and Learning Executive Function | Self-Report: **1.Multidimensional Fatigue Inventory (MFI-20)** Evaluates different aspects of fatigue. **2.EORTC QLQ-C30** Measures quality of life in cancer patients.**3.Beck Depression Inventory (BDI-II)** Assesses severity of depressive symptoms across emotional, | **Executive functioning-** most affected domain, with 41% impaired: 27% on TMT-B, 14% on CANTAB-IED, 10% on CANTAB-SOC, and 2% on the Stroop interference task. **Learning and memory-** 28% showed impairments, including 22% on the first CVLT trial, 10% on short re | Survivors reported significantly more frequent and severe subjective experiences of cognitive impairment and fatigue compared to controls, as measured by the FACT-Cog and MFI-20 respectively. However, severe depression and anxiety were infrequently report |  |  |
| Tan et al. 2020 | Longitudinal | 91 | Male=49 (53.8%), Female=42 (46.2%) | 15 to 39 years | 0.8 months, median IQR=0 to 1.8 months |  | mean=28.4 years (SD=6.7) |  | Mixed [Lymphoma=30 (33%)] | Chemotherapy=52 (57.1%), Radiotherapy=25 (27.5%) | Self-Report: Functional Assessment of Cancer Therapy-Cognitive Function (FACT-Cog) | Perceived Cognitive Impairment | Distress thermometer (DT)- brief screening tool that measures distress, Rotterdam Symptom Checklist (RSCL)- assess symptom burden | 34.1% of participants reported CRCIat least once during the study period, Similar to FACT-Cog, a proportion of participants also indicated problems with memory or concentration on the DT problem checklist at each time point | Female gender (P = .02), Indian ethnicity (P < .01), current smokers (P < .01), anxiety/depressive symptoms (P < .01) and fatigue (P < .01) were found to be associated with poorer cognitive function among AYAs | assessed at four time points over 1 year: (a) baseline at recruitment (T1); (b) 1 ± 0.5 month later (T2); (c) 6 ± 2 months later(T3); and (d) 12 ± 2 months later (T4) |  |
| Dewar et al. 2021 | Cross sectional | 2646 AYA survivors | Male=486 (18.4%), Female=2160 (81.6%) | 15 to 39 years | at least 10 years |  | 55 years, median IQR=47 to 66 years | 228,029 (no history of cancer control group) | Mixed [Blood/leukemia/lymphoma=156 (5.9%)] | NR (the NHIS database does not include details on cancer treatment) | NHIS Functioning and Disability Questionnaire | Self-Reported Cognitive Impairment | Kessler Psychological Distress Scale (K6) | Cognitive dysfunction was more prevalent in cancer survivors (24.8%) than in the general population (15.3%). Among affected AYA survivors, 28.5% had memory issues, 12.0% had trouble concentrating, and 59.5% reported both. In the non-cancer group with cognitive dysfunction, 30.2% had memory issues, 14.1% had difficulty concentrating and 55.6% reported both. | The prevalence of having psychological distress was 24.9% (95% CI 22.1–28.0%) in cancer survivors as compared to 14.3% (95% CI 14.0–14.6%) in the general population |  |  |
| Mariegaard et al. 2021 | Cross sectional | 116 | Male 50%, Female 50% | NR |  | mean=29.6 months | 20 to 60 years, median 39.5 years (mean=40.3 years) |  | Hodkin Lymphoma (57%), Diffuse Large B Cell Lymphoma (43%) | Chemotherapy (94%), Radiation (66%) | Self-report questionnaire: Behavior Rating Inventory of Executive Function-Adult (BRIEF-A) | Executive function: Inhibition, Shifting, Emotional Control, Self-monitoring, Initiation, Working Memory, Planning/Organization, Task-Monitoring and Organization of Materials. | Hospital Anxiety and Depression Scale (HADS)- to measure anxiety and depression symptoms, Perceived Stress Scale (PSS), Pittsburgh Sleep Quality Index (PSQI), European Organisation for The Research and Treatment of Cancer Quality of Life Questionnaire-Cor | 39% reported executive impairment [(n = 45) scored > 65 on at least one subscale of the BRIEF-A], Working Memory was the domain most frequently reported as impaired (24%) | Survivors reporting impaired executive functioning reported worse mental health (ps < .001) than survivors reporting non-impaired executive functioning. A larger proportion of the impaired group had received a high chemo dose compared to the non-impaired |  |  |
| Mellblom et al. 2021 | Retrospective, Cross sectional | 359 blood cancer participants | Female=165 (45.9%), Male=194 (54.1%) | 19 to 39 years mean= 29.1 years(Leuk, 30.8 years (NHL) | average 17 years |  | mean= 48.5 years NHL, 46.6 years Leuk |  | Mixed [NHL=225, Leuk=134] | Systemic (single chemotherapy or other systemic treatments) and Multimodal (systemic treatment with surgery, radiotherapy, or stem cell transplantation) | self-report NORCAYACS questionnaire | **Self-reported late effects** (inclusive of concentration and memory problems) and follow up care |  | Survivors of NHL reported the highest number of late effects, of which 33% NHL reported 5 or more late effects. 33.3% of NHL and 35.1% Leuk survivors reported concentration and memory problems | Among the survivors who had received multimodal treatments, 55% reported 3 or more late effects compared with 30% for systemic treatment, 11% for local treatment, and 2%for minimal surgery (data inclusive of other cancers as well) |  | Risk of biased sample and non-response bias |
| Brock et al. 2022 | Prospective, Longitudinal | 502 | Male=127 (25.3%), Female=375 (74.7%) | 18–39 years at diagnosis, median=29.9 years | <4 years, mean=12.15 months |  | NR |  | Mixed [HL=92 (18.3%), NHL=32 (6.4%), Haematological=35 (7%)] | **Chemotherapy** (includes radio chemotherapy)=**381 (75.9%**), **Radiotherapy** (includes radio chemotherapy and nuclear therapy)=**241 (48%**) | Cognitive impairments (Copenhagen Psychosocial Questionnaire, COPSOQ) |  | survey: Work ability (Work Ability Index, WAI-r), self-assessment: Perceived Adjustment to Chronic Illness Scale (PACIS) | Approximately 20% of AYA cancer survivors reported cognitive impairments at both timepoints (t1 and t2), indicating that these difficulties are persistent over time [n = 88 (18%) reported CRCI at t1 and n = 82 (16%) at t2]. | Work ability improved significantly from t1 (mean = 6.0) to t2 (mean = 6.8), p < 0.001. However, 57% of participants still reported reduced work ability at t2, down from 76% at t1. CRCI affected significantly more women than men and was the strongest pred | two time points (t2 = 12 months after t1) | Risk of biased sample |
| Baratto et al. 2024 | Prospective, single center pilot study | 10 |  | NR |  | mean=3.5 ± 1.5 months | 9–23 years (mean ± SD, 17.4 ± 4.7 y) | NR | Sarcoma=5, Lymphoma=4, Leukemia=1 | Chemotherapy [n=10 intravenous HDMTX, n=5 intrathecal methotrexate] | Wechsler Abbreviated Scale of Intelligence, Second Edition (WASI-II), Wide Range Assessment of Memory and Learning, Second Edition (WRAML-2), Delis–Kaplan Executive Function System (D-KEFS) | Overall intellectual, memory and executive functioning | PET/MRI Scan | Nine of 10 patients showed at least one negative z score on neurocognitive testing (mean no. of impaired tests = 3; range = 1–7), indicating deficits in cognitive function. The most frequently impaired domain was visual memory (WRAML-VIS: 8 patients with impairment, 6 for WASI–verbal comprehension index, and 4 for DKEFS-TM4) | Cognitive impairment correlated with lower brain activity (SUV mean) in the prefrontal cortex and cingulum, linked to poorer executive function (DKEFS-TM4). Cerebral blood flow (CBFmean) was lower in the hippocampus and correlated with both executive funct |  |  |
| Williams et al. 2024 | Retrospective, Cross sectional | 197 | Male=95 (48.2%), Female-102 (51.8%) | 3 to 26 years, Median=14.84 years | median=20.5 years |  | 22 to 64 years, median= 35.41 years (SD=8) | 199 (age-,sex-and race/ethnicity frequency-matched community controls) | Hodgkin Lymphoma | Chemotherapy and Radiation (chest and neck) | Woodcock-Johnson III Tests of Achievement, Trail Making Test A, Trail Making Test B, Conners' Continuous Performance Test (2nd Ed), Wechsler Abbreviated Scale of Intelligence, Wechsler Adult Intelligence Scale (4th Ed), California Verbal Learning Test, 2n | intelligence/academics, attention, processing speed, memory, and executive function | physical examination, laboratory, echocardiography, and pulmonary function testing | Hodgkin lymphoma survivors showed significantly poorer performance than controls across multiple cognitive domains. | Elevated hs-CRP (≥3 mg/L) was associated with worse attention, processing speed, memory, and executive function (p < 0.05). IL-6 was linked to lower visuomotor processing speed (p = 0.046), and malondialdehyde was associated with poorer attention and visual | Within 3 days of neurocognitive testing, participants had a physical examination, laboratory, echocardiography, and pulmonary function testing | 40 survivors (20%) had subsequent malignancies |
